# Exposure to insulating materials and risk of coronary artery diseases: a cross-sectional study

**DOI:** 10.3389/fpubh.2023.1235189

**Published:** 2023-08-07

**Authors:** Subhabrata Moitra, Ali Farshchi Tabrizi, Fadi Khadour, Linda Henderson, Lyle Melenka, Paige Lacy

**Affiliations:** ^1^Alberta Respiratory Centre and Division of Pulmonary Medicine, Department of Medicine, University of Alberta, Edmonton, AB, Canada; ^2^Synergy Respiratory and Cardiac Care, Sherwood Park, AB, Canada

**Keywords:** asbestos, chronic respiratory disease, Framingham risk score, worker's health, occupational exposure

## Abstract

**Background:**

Although previous reports link exposure to insulating materials with an increased risk of mesothelioma and chronic respiratory diseases, studies evaluating their associations with the risk of coronary artery diseases (CAD) are lacking.

**Aims:**

We aimed at evaluating the associations between exposure to insulating materials and the 10-year risk of CAD among insulators.

**Methods:**

In this cross-sectional study, we recruited 643 adults (≥18 years), full-time insulators from the Local 110 Heat and Frost Insulators and Allied Workers Union in Edmonton, Alberta. We obtained demographic information, personal and family history, and job-exposure history, including experience (years) and types of exposure to insulating materials. Clinical profiling including Framingham risk scores (FRS) was assessed.

**Results:**

Of all insulators, 89% were men (mean ± SD age: 47 ± 12 years), 27% had a parental history of cardiac diseases, and 22% had a comorbid chronic respiratory disease. In total, 53% reported exposure to asbestos, while 61, 82, and 94% reported exposure to ceramic fibers, fiberglass, and mineral fibers, respectively. In single-exposure multivariable regression models adjusted for experience, marital status, and body mass index (BMI), asbestos was found to be associated with higher FRS (*β*: 1.004; 95%CI: 0.003–2.00). The association remained consistent in multi-exposure models and a higher association was found between asbestos exposure and FRS among insulators with comorbid chronic respiratory disease.

**Conclusion:**

Our study demonstrates that apart from cancer and chronic respiratory diseases, asbestos exposure may also have a cardiac effect, thus warranting the need for systematic surveillance to protect workers from the adverse effects of these materials.

## Introduction

Several new man-made materials, often known as man-made vitreous fibers (MMVFs), aerogels, carbon fibers, mineral fibers, and refractory ceramic fibers (RCFs), have been introduced as potential insulating materials after many countries have imposed a ban on the use of asbestos. Most of these fibers also possess significant health hazards, particularly affecting the respiratory system that led to numerous adverse conditions such as chronic chest infection, carcinoma, adverse pleural conditions, and to some extent, obstructive and interstitial changes in the lungs (1–8). However, despite the ban on asbestos use, workers, particularly construction workers and insulators, are often exposed to it, for example, during the demolition or renovation of old construction and insulations (9–11).

Although numerous significant information about the toxicity and possible adverse respiratory health effects of these materials is available from historic and contemporary clinical and public health studies, a majority of those largely focused on respiratory and cancer-related outcomes (6, 9–21), and little is known about their involvement in other target organs, e.g., cardiovascular health (22–24). While some previous studies indicated possible cardiovascular health effects of occupational asbestos exposure, most of them have been primarily investigated post-mortem, i.e., the involvement of asbestos in cardiovascular disease-related mortality could only be established retrospectively (23, 25). Nevertheless, several reports showed no significant cardiovascular effects regarding asbestos exposure (26). Finally, many of those studies also reported the co-occurrence of malignancies and other chronic diseases because of asbestos exposure; therefore, whether such adverse cardiovascular health conditions were caused by asbestos or the consequences of other health conditions could not be made clear. Similarly, literature on the possible cardiovascular health effects of MMVFs is scarce and produced mixed evidence. However, whether exposure to such materials is associated with the risk of coronary artery disease (CAD) has not been systematically investigated.

In this study, we aimed to investigate whether occupational exposure to insulating materials (asbestos, aerogels, calcium silicate, carbon fibers, fiberglass, mineral fibers, and RCFs) was associated with an increased risk of CAD in insulators.

## Methods

In this cross-sectional study, we investigated 843 unionized insulators of the Local 110 Heat and Frost Insulators and Allied Workers Union in Edmonton, Alberta recruited between 2011 and 2014. All participants were screened at Synergy Respiratory Care Clinic, Sherwood Park, Alberta. Details of the study design, inclusion and exclusion criteria, and methodologies have been reported elsewhere (7, 8). In brief, participants of at least 18 years of age, employed full-time as insulators, and of sound mental and cognitive ability at the time of screening were included in the study. We excluded those who were <18 years of age and trainees without a history of insulation material exposure. All participants were administered a questionnaire containing items of demographic profile, personal and family history (smoking history and pack-years, alcohol consumption, parental history of any cardiac diseases, and frequency of weekly physical activity), and detailed job-exposure history including experience (years), types of exposure to insulating materials, and the use of personal protective equipment (PPE) at work for each of the materials (such as aerogels, asbestos, calcium silicate, carbon fibers, fiberglass, mineral fibers, and RCFs).

Detailed clinical profiling of the participants was performed at the clinic that included an assessment of any current respiratory and cardiac conditions, such as hypertension and chronic respiratory diseases (CRDs), and any previous incidents of cardiorespiratory or metabolic conditions such as chest pain/angina, heart attack, heart failure, heart failure, cardiac catheterization, coronary bypass surgery, angioplasty, atrial fibrillation, lung cancer, diabetes mellitus, or any other acute conditions. Current medication status was obtained from questionnaires and was verified from the participants' health records. A venous blood sample was collected, and the plasma lipid profiles (cholesterol, triglyceride, and high- and low-density lipoproteins) were analyzed. Framingham risk score [FRS; the 10-year risk of manifesting clinical cardiovascular diseases such as CAD, stroke, peripheral vascular diseases (PVD), chronic heart failure (CHF), and cardiac death] was calculated according to the previously established formulae (27). All data were anonymized before further analysis.

Data were presented as mean [standard deviation (SD)], median [interquartile range (IQR)], or frequency (%) for continuous, ordinal, and categorical variables, respectively. We first tested the bivariate relationships between each of the exposures (yes/no) and FRS using Student's *t*-tests. We then constructed univariable (unadjusted) and multivariable (adjusted) linear regression models for each exposure and FRS as the following:


$$\begin{array}{l} \hat{Y}=\beta_{0}+\beta_{1}X_{1}+\beta_{2}X_{2}+\beta_{3}X_{3}+\ldots +\beta_{n}X_{n}+\varepsilon \end{array}$$

where *Ŷ* is the predicted or expected value of the dependent variable, *β*_0_ is the Y-intercept, *β* is the regression coefficient of the independent variable of *X, n* is the number of independent variables, and ε is the residual.

Ethnicity, education, marital status, body mass index (BMI), years of exposure to insulation work, and the use of PPE were tested as potential confounders. As age, sex, and smoking status were adjusted while calculating the FRS, they were not further considered confounders. Models were constructed in step-forward and step-backward algorithms and only marital status, body mass index (BMI), and years of exposure to insulation works were retained in the final models based on the Akaike information criterion (AIC) for model selection (28) as per the following equation:


$$\begin{array}{l} AIC=2k-2ln\left(\hat{L}\right) \end{array}$$

where *k* is the number of estimated parameters in the model and $\hat{L}$ is the maximum value of the likelihood function for the model.

We also performed several secondary analyses. First, we created a multi-exposure linear model (taking all the exposures in a single model) and tested the associations with FRS as the exposure variables did not demonstrate collinearity among them (variance inflation factor, VIF < 2). Second, we stratified the single-exposure models by sex and tested the pair-wise differences of the coefficients using the Chow test (29). Finally, we tested potential effect modification by alcoholic drink per month (<1 vs. ≥1 per month), any physical activity other than regular work (no/yes), parental history of cardiac disease (no/yes), and any CRDs (no/yes). All analyses were conducted using a complete case approach in Stata V.17 (StataCorp, College Station, TX, USA), and a *p*-value of <0.05 was considered statistically significant.

This study has been conducted as per the Declaration of Helsinki and is compliant with the Strengthening the Reporting of Observational Studies in Epidemiology (STROBE) guidelines (30) and approved by the Health Research Ethics Board of Alberta (HREBA.CTC-17-0067) and Health Research Ethics Board (Pro00079792), University of Alberta. All participants provided signed informed consent forms before taking part in the study.

## Results

Of those 843 insulators who were screened, we further excluded those with a current or previous clinical diagnosis of one or more conditions such as chronic chest pain/angina (*n* = 119), heart attack (*n* = 20), heart failure (*n* = 3), cardiac catheterization (*n* = 22), coronary bypass (*n* = 8), angioplasty (*n* = 21), atrial fibrillation (*n* = 27), mesothelioma (*n* = 1), or other chronic or acute cardiopulmonary conditions (*n* = 118). We finally recruited 643 insulators for this analysis of which 571 (89%) were men with a mean age (SD) of 47 (12) years. In total, 78% were Caucasians, 67% were ever smokers, 27% reported a family history of cardiac diseases, and 22% had comorbid CRDs (Table 1). Exposure to different insulating materials ranged between 39% (aerogels) and 94% (RCF), and 53% of the workers reported having exposure to asbestos at workplaces. The mean (SD) FRS was 7.3 (6.6).

**Table 1:** Demographic profile, exposure history, and clinical profile of the insulators.

	***N* = 643**
Age (years), mean (SD)	47 (12)
Sex (male), *n* (%)	571 (89)
BMI (Kg/m^2^), mean (SD)	30 (15)
Ethnicity (Caucasian), *n* (%)	501 (78)
**Education**, ***n*** **(%)**	
Grade school	17 (3)
High school	77 (12)
Trade school	302 (47)
College/university	247 (38)
Marital status (married), *n* (%)	407 (63)
**Smoking status**, ***n*** **(%)**	
Ever smoker	429 (67)
Overall pack-years, median (IQR)	5 (0–17)
Any chronic respiratory diseases, *n* (%)	141 (22)
**Alcohol consumption**, ***n*** **(%)**	
< 1 drink per month	77 (12)
**Physical activity**, ***n*** **(%)**	
Never	297 (46)
1–3 times/week	209 (33)
4–6 times/week	65 (10)
Daily	72 (11)
Parental history of cardiac disease, *n* (%)	169 (27)
**Exposure variables**	
Years of exposure in insulation work, median (IQR)	13 (4–28)
**Exposure to insulating materials**, ***n*** **(%)**	
Aerogels	253 (39)
Asbestos	343 (53)
Calcium silicate	552 (86)
Carbon fibers	370 (58)
Ceramic fibers	393 (61)
Fiberglass	525 (82)
Mineral fibers	603 (94)
**PPE used for**, ***n*** **(%)**	
Aerogels	227 (35)
Asbestos	181 (28)
Fiberglass	282 (44)
Mineral fibers	447 (70)
**Cardiometabolic variables**	
Systolic blood pressure (mmHg), median (IQR)	124 (115–132)
Clinically diagnosed hypertension, *n* (%)	117 (18)
Medications for hypertension, *n* (%)	91 (14)
**Lipid profile (mg/dL), mean (SD)**	
Cholesterol	195.2 (41.5)
Triglyceride	140.2 (90.2)
HDL	50.8 (14.6)
LDL	118.1 (36.0)
Framingham risk score, mean (SD)	7.3 (6.6)

Data presented as mean [standard deviation (SD)], median [interquartile range (IQR)], or frequencies (%) as described in the text.

BMI, body mass index; HDL, high-density lipoprotein; LDL, low-density lipoprotein; PPE, personal protective equipment.

**Figure 1:**
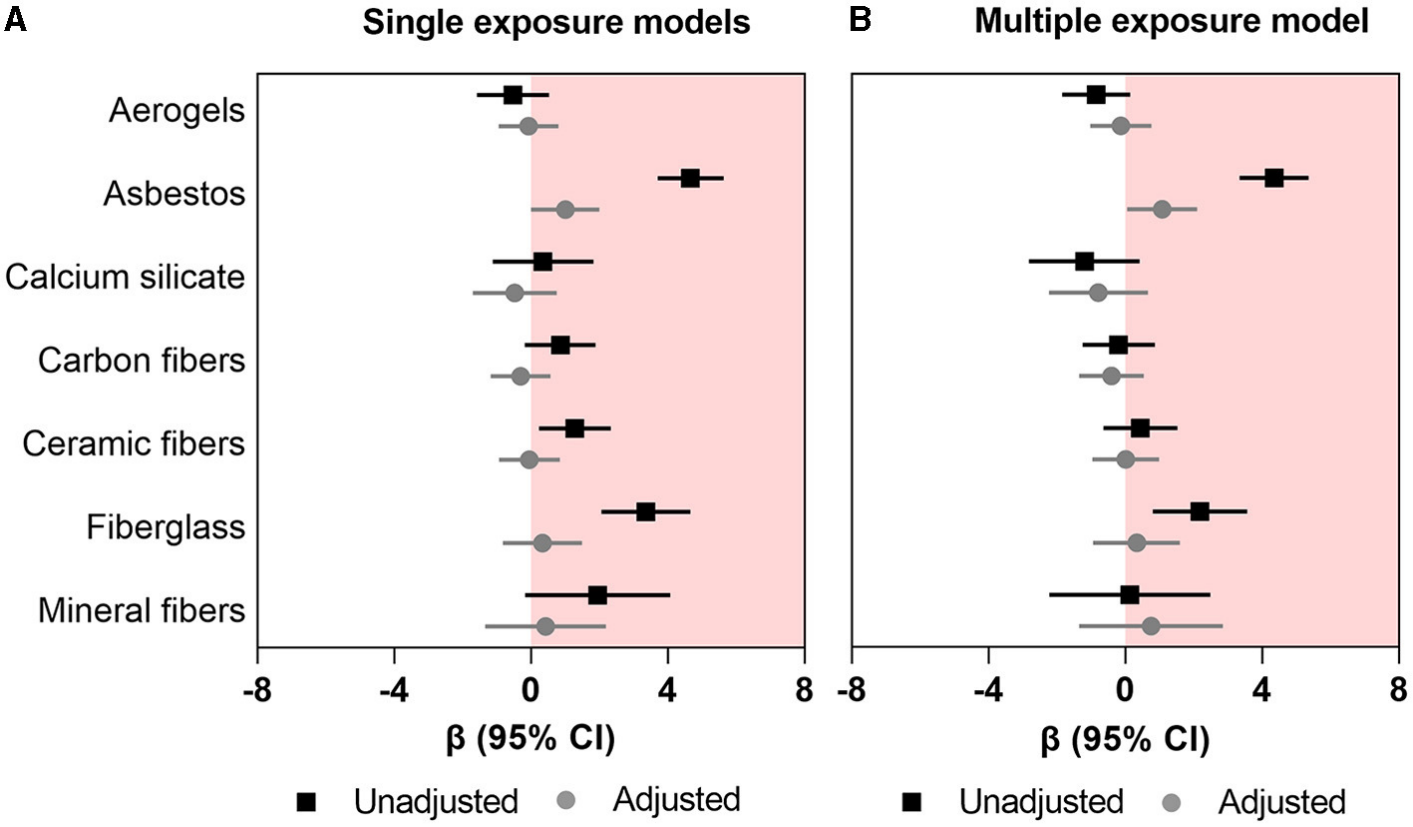
Association between occupational exposure to mineral fibers and Framingham risk score. **(A)** Single exposure models **(B)** multiple exposure models. Data presented as regression coefficient (marker) and 95% confidence interval (error bars) adjusted for marital status, BMI, and years of exposure in insulation works.

In single-exposure univariable (unadjusted) models, asbestos, ceramic fibers, and fiberglass (*β* range: 1.28–4.66; all *p*-values < 0.05) were associated with higher FRS (Figure 1A; Supplementary Table 1); however, in the adjusted (multivariable) models, only asbestos retained a significant association with higher FRS (*β*: 1.004; 95%CI: 0.003–2.00). In the multi-exposure model, only asbestos remained associated with higher FRS (*β*: 1.08; 95%CI: 0.05–2.10) (Figure 1B; Supplementary Table 1).

**Figure 2:**
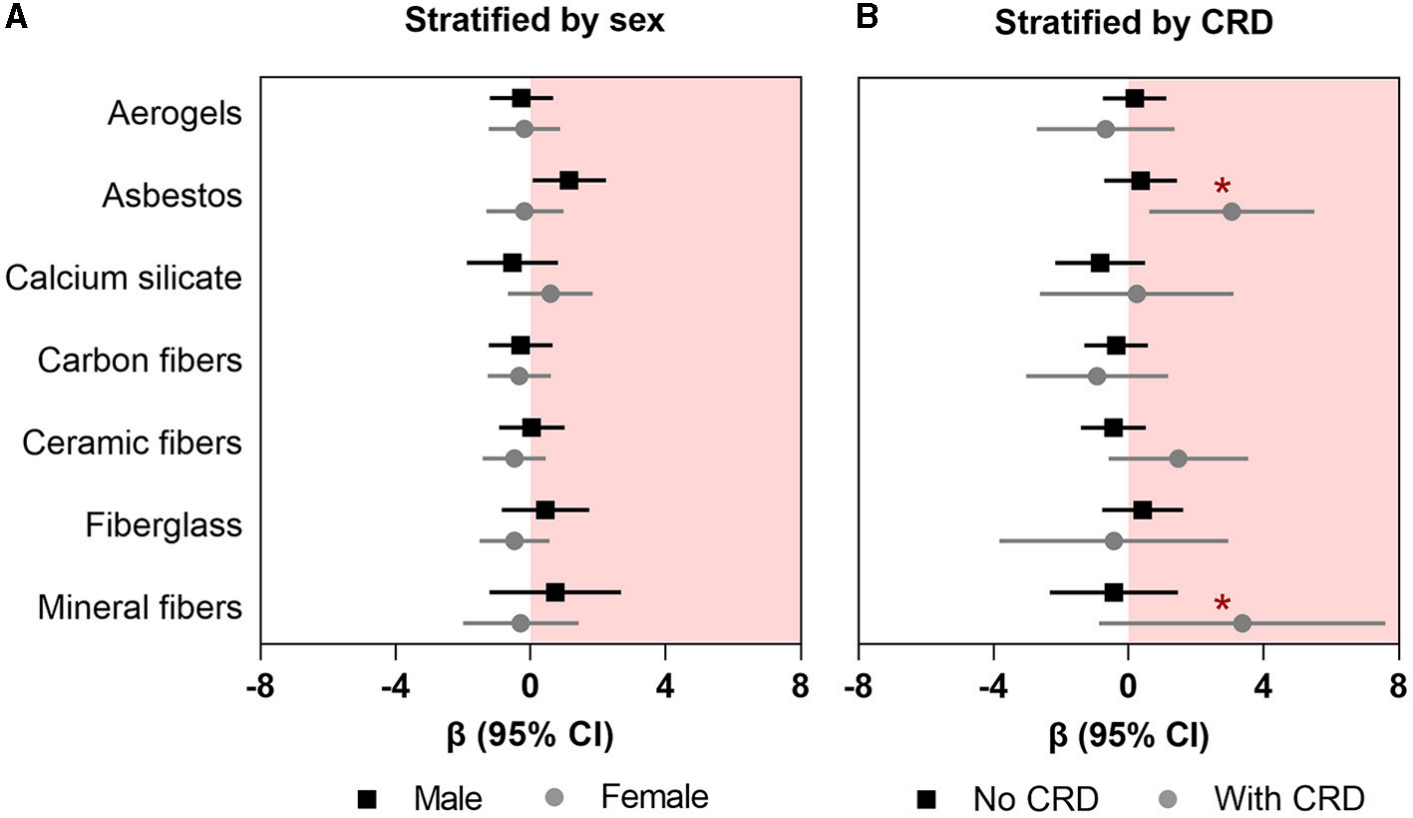
Association between occupational exposure to mineral fibers and Framingham risk score **(A)** stratified by sex and **(B)** effect modification by any chronic respiratory disease (CRD). Data presented as regression coefficient (marker) and 95% confidence interval (error bars) adjusted for marital status, BMI, and years of exposure in insulation works. *Indicates the *p*-values for interaction that are significant at *p* < 0.05.

In the secondary analyses, we observed a higher association between asbestos exposure and FRS in male insulators (*β*: 1.14; 95%CI: 0.06–2.23) than their female counterpart (*β*: −0.18; 95%CI: −1.31–0.97); however, the difference between the coefficients was not significant (chi-squared p-value for Chow test = 0.13) (Figure 2A; Supplementary Table 2). Furthermore, we found that the association between asbestos exposure and FRS was significantly higher among the insulators with comorbid CRDs (*β*: 3.05; 95%CI: 0.61–5.50) than those without any comorbid CRDs (*β*: 0.36; 95%CI: −0.72–1.43; *p*-value for interaction: 0.007) (Figure 2B; Supplementary Table 3). We did not observe any clinically important influence of other effect modifiers (alcoholic drink per month, any physical activity other than regular work, or parental history of cardiac disease) on the associations between insulating materials and FRS (Supplementary Tables 4–6).

## Discussion

To the best of our knowledge, this is the first study assessing the relationship between several types of insulating materials and the 10-year predictive risk score for CAD in a group of workers. We observed that of all insulating materials, only asbestos exposure was associated with a higher FRS. This association was stronger among male subjects and those with comorbid CRDs. We did not find any associations between other insulating materials and FRS or any influence of possible behavioral or genetic factors such as alcohol consumption, physical activity, or a family history of cardiac diseases.

Our findings of asbestos-associated increased projected risk of cardiovascular diseases extend the observations of a recent meta-analysis where Rong et al. (24) demonstrated the pooled standardized mortality ratio (SMR) estimate for cardiovascular-related diseases of 1.11 (95% CI: 1.01–1.22), indicating a significant association between asbestos exposure and an increased risk of cardiovascular-related diseases in exposed workers. Nevertheless, despite numerous post-mortem studies on asbestos exposure and cardiovascular disease-related mortality, the mechanisms of asbestos exposure-associated cardiovascular diseases have not been fully understood. One possible explanation could be the physiological alteration of the heart and the vasculature due to asbestos exposure, as a previous study showed pericardial thickening in asbestos-exposed workers (31). Another possible explanation for the increased risk of cardiovascular diseases could be the inflammatory effects of asbestos on the cardiopulmonary circuit, leading to a sustained accumulation of inflammatory mediators, and an upsurge in oxidative stress, all of which possibly help in the development of atherosclerotic plaques (32). While some animal studies support this hypothesis of free radical production, inflammation in coronary arteries, and vascular and thrombogenic effects of asbestos exposure (33–35), human data on the mechanisms are unavailable and difficult to obtain.

Our findings of the roles of other insulating MMVFs on FRS are similar to the meta-findings of previous studies (36–42), which did not indicate any significant associations between MMVFs and cardiovascular diseases. Although a relatively recent study reported a higher increased risk for ischemic heart disease among Swedish construction workers, the study used a job-exposure matrix that considered a wide array of exposures such as dust, gas, fumes, and other particulate matters (43). Therefore, it was not clear whether the increased risk of cardiovascular diseases was specifically attributed to exposure to MMVFs.

We found that the association between asbestos exposure and FRS was higher among those with CRDs than those without CRDs. Previous data have shown that asbestos exposure was associated with a higher prevalence of CRDs such as chronic obstructive pulmonary disease (COPD) (7, 44). It is well-established that patients with comorbid CRDs are at a higher risk of having CAD than those without CRDs (45–51). Therefore, insulators with a history of asbestos exposure and comorbid CRDs were at a higher risk of developing CAD; however, the cross-sectional nature of the study does not allow us to infer whether CRDs had an additive effect on the predicted risk of CAD.

One of the major strengths of this study is its novel approach to assessing a chronic and probable detrimental cardiological effect of the insulating materials that have not been previously investigated. Second, we considered a wide range of insulating materials and tested their individual and cumulative associations with the FRS. Finally, we performed several secondary analyses to test the plausible influence of other potential lifestyle and clinical factors on the association between insulating materials and FRS, which highlighted the significant influence of comorbid CRDs. Of note, we calculated FRS based on a previously reported clinical practice report (27), which describes the lower age for FRS calculation as 20 compared to other modified versions. As our participants belonged to a high-risk group for occupational exposure, we did not add the age constraint as described in other versions of the FRS calculation which could have undermined a vulnerable population. However, the study has some limitations as well. As the current analysis is cross-sectional in design, we could not determine any causal association between exposure to asbestos and incident of CAD. Second, we only had information on whether the insulators were exposed to insulating materials or not, and we could not measure the level of exposure to each insulating material, for example, the specific type of asbestos or other fibers, or determine the cumulative exposure index. Similarly, information about PPE (duration of use, brand, or specifications) was not available. However, adjusting the models for PPE did not significantly alter the magnitude of the estimates, which means that the PPE did not provide substantial protection to the workers from exposure. Moreover, we could not perform any detailed biochemical studies, such as oxidative stress assessment, which could have been useful to explain the cardiological impacts of these insulating materials. Finally, we did not have detailed information about other potential workplace exposures such as physical and chemical agents, heat or cold exposure, and other behavioral and socioenvironmental triggers, which could also impart adverse health effects. Thus, these factors also need to be considered in future studies.

Our findings add substantially to the current understanding of asbestos-related health effects and highlight an important health concern of asbestos exposure by recommending more holistic surveillance of the workplace. It also warrants amending adequate protective measures and administering more frequent and rigorous monitoring of health to minimize the risk of work-related health hazards. Asbestos-related diseases are often diagnosed years after such exposures due to their long latency. Therefore, physicians need to consider possible work-related exposures to hazardous materials which might influence the development of such diseases.

We may conclude that occupational exposure to insulation materials, particularly asbestos, is associated with a higher risk of CAD in insulators. The association was found higher among those who have comorbid CRDs such as asthma or COPD possibly due to the cardiologic effects of CRDs; however, we could not confirm the associations from an etiological perspective. While the adaptation of less hazardous insulating materials and the use of proper PPE are recommended, a regular health monitoring and workplace surveillance program must be implemented for diagnosis.

## Data availability statement

The datasets presented in this article are not readily available because the provincial law does not allow public dissemination of primary health data. Requests to access the datasets should be directed to PL, placy@ualberta.ca.

## Ethics statement

The studies involving human participants were reviewed and approved by the Health Research Ethics Board of Alberta (HREBA.CTC-17-0067) and the Health Research Ethics Board (Pro00079792), University of Alberta. The patients/participants provided their written informed consent to participate in this study.

## Author contributions

LM: conceptualization and funding acquisition. FK and LM: methodology and validation. SM: formal analysis, writing—original draft preparation, and visualization. LH, FK, and LM: investigation. PL and LM: resources and supervision. AT: data curation. LH and PL: project administration. All authors: writing—review and editing. All authors have read and agreed to the published version of the manuscript.
